# Supplementary figures and images for: Rapid ER remodeling induced by a peptide–lipid complex in dying tumor cells
Source: Life Sci Alliance. 2025 Mar 25;8(6):e202403114. doi: 10.26508/lsa.202403114 (PMC11938384; doi:10.26508/lsa.202403114)

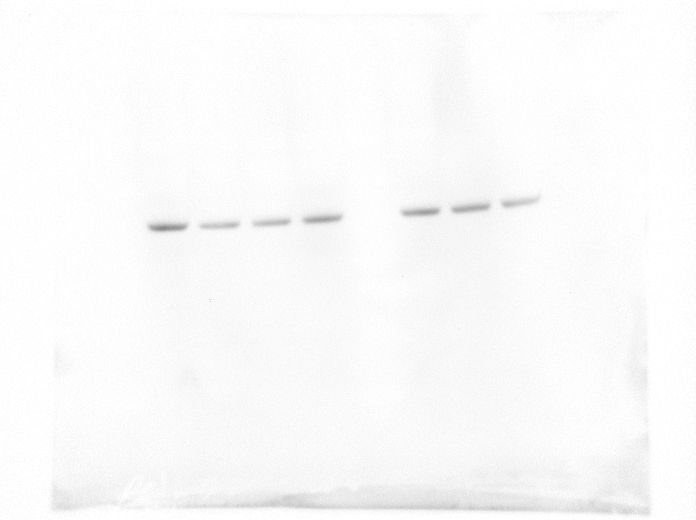

Supplement: Supplementary file 1 [file LSA-2024-03114_SdataFS21.1.tif]

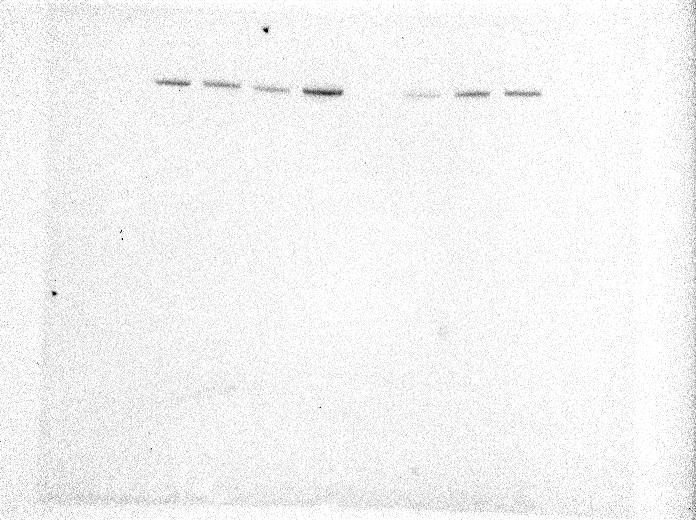

Supplement: Supplementary file 2 [file LSA-2024-03114_SdataFS21.2.tif]

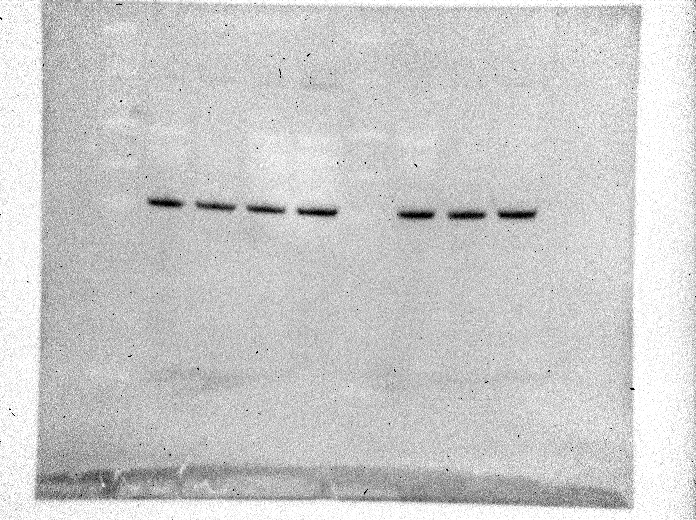

Supplement: Supplementary file 3 [file LSA-2024-03114_SdataFS21.3.tif]

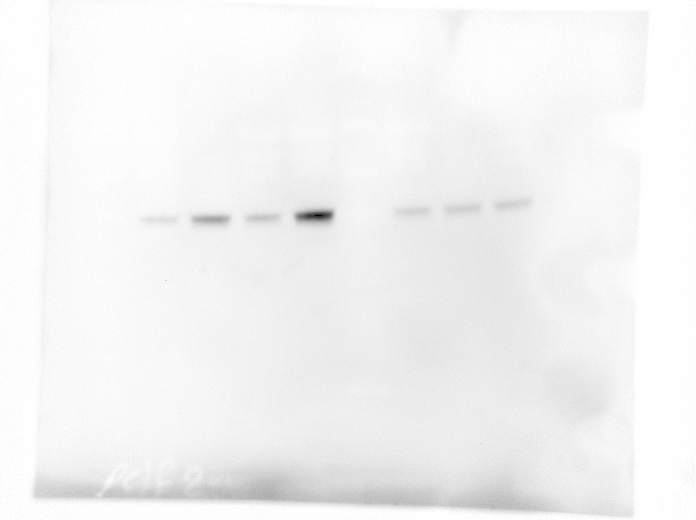

Supplement: Supplementary file 4 [file LSA-2024-03114_SdataFS21.4.tif]
